# Relationship between Biological, Training, and Physical Fitness Variables in the Expression of Performance in Non-Professional Runners

**DOI:** 10.3390/sports9080114

**Published:** 2021-08-23

**Authors:** Mabliny Thuany, Thayse Natacha Gomes, Marcos B. Almeida

**Affiliations:** 1 CIFI2D, Faculty of Sports, University of Porto, 4200-450 Porto, Portugal; mablinysantos@gmail.com; 2 Department of Physical Education, Federal University of Sergipe (UFS), São Cristóvão 49100-000, SE, Brazil; mb.almeida@gmail.com; 3 Post-Graduation Program of Physical Education, Federal University of Sergipe, São Cristóvão 49100-000, SE, Brazil

**Keywords:** physical fitness, running, performance

## Abstract

Sports performance is a multifactorial trait that can be associated with individual and environmental characteristics. In this study, the sample comprised 35 male runners, enrolled in the “InTrack” project. Information regarding variables related to runners’ training was obtained via an online questionnaire, while anthropometric and body composition variables, as well as physical fitness components (muscular power, isometric strength, local muscular endurance, agility, and aerobic capacity) were measured, and a global physical fitness score (based on physical fitness components measured) was computed. The Weltman test (3200 m) was used to estimate runners’ pace and their stride frequency. Linear regression was used, taking the running pace as dependent variable. The final model, comprising biological, physical fitness, spatiotemporal, and training variables, explained 86% of the running performance variance. Muscular power (β = −1.02; 95% CI = (−1.69)–(−0.35)), abdominal muscle endurance (β = −4.81; 95% CI = (−7.52)–(−2.10)), isometric strength (β = −422.95; 95% CI = (−689.65)–(−156.25)), global physical fitness (β = 27.14; 95% CI = 9.52–45.03), and stride frequency (β = −2.99; 95% CI = (−4.29)–(−1.69)) were significantly associated with performance, meaning that better results in tests and increasing the stride frequency leads to better performance. Individual characteristics and physical fitness components were demonstrated to be significant predictors for running performance.

## 1. Introduction

The increasing number of runners around the world [1] has led researches and coaches to investigate factors related to performance [2,3], aiming to understand the predictors that could differentiate runners into distinct groups. In this context, aerobic characteristics and physiological factors, such as VO_2máx_ [4], anaerobic threshold [5], and running economy [6], have been largely investigated [7,8].

Considering that road running is primarily performed in outdoor spaces, runners are usually exposed to some constraints, related to both built or natural environments (such as different surfaces, going uphill, sidewalks) or opponents [7]. Thus, athletes must provide a quick response to these constraints which, for example, may require agility, muscular resistance, and/or lower body strength. These components have been considered key factors for running performance, due to their influence in movement efficiency [9]. However, little is known about the role of these anaerobic components to explain (and to differentiate) runners’ performance [10], given that most published studies have examined the association between strength and power among short distance runners (10–300 m) [11]. When these components were investigated among long distance runners, a relationship was observed between jump ability and running performance [12], and also an association between isometric muscle strength and lower body flexibility with the performance of male and female recreational marathon runners, respectively [10,13].

Notwithstanding the relevance of these studies, it is important to highlight that the majority did not investigate different physical fitness components simultaneously, but focused on understanding the relevance of some of them separately in running performance. Moreover, the measurement of the components usually requires expensive equipment/techniques, which are not accessible for most researchers (and coaches). Furthermore, in training programs, runners are exposed to several methodological training approaches, which can involve strength, coordination, and plyometric and functional training [14,15,16,17,18], which are proposed with the aim to develop several physical capacities such as agility and muscular endurance. Since performance is a multifactorial trait [19], and training plays an important influence in race time, which allows the identification of the best runners [20,21], it seems necessary to consider the simultaneously influence of distinct physical fitness variables in its expression. So, the purpose of this study was to identify the association between physical fitness, and biological and training variables with performance among non-professional runners.

## 2. Materials and Methods

### 2.1. Design and Sample

The participants were recruited from the “InTrack” project [22], a study aiming to identify factors associated with road running performance. The sample comprised 35 male runners, aged 18–60 years, invited through social media and runners’ coaches’ contact. To be eligible to participate, runners answered an online questionnaire, were over the age of 18 years, and were evaluated in all study tests. The study was conducted according to the guidelines of the Declaration of Helsinki, and approved by the Ethics Committee of the Federal University of Sergipe, Brazil (protocol No. 3.558.630).

### 2.2. Data Collection and Procedures

Participants were measured for all variables in a unique day, in the morning (from 8:00 a.m. to 10:00 a.m.) or in the afternoon (from 3:00 p.m. to 6:00 p.m.), according to their choice/availability.

### 2.3. Training Variables

It was obtained information about training frequency/week, volume/week, and practice time through an online questionnaire (via Google Forms), named “Profile characterization and associated factors for runner’s performance” [23]. 

### 2.4. Anthropometric and Body Composition Variables

Height, weight, and skinfold thickness measurements followed procedures described by the International Society for the Advancement of Kinanthropometry [24]. Subjects stood up in a portable stadiometer (Slim Fit, Brazil, 0.1 cm scale) for height, and in a digital scale (TechLine, São Paulo, Brazil, 0.1 kg scale) for body mass measures, being barefoot and wearing light clothes. Triceps, subscapular, iliac crest, and medial calf skinfolds were measured on the right side of the body using a Calliper (Sanny, São Paulo, Brazil, 0.1 mm scale). To estimate the body density and body fat percentage (body fat%), Petroski [25] and Siri [26] equations were used, respectively.

### 2.5. Physical Fitness Variables

Physical fitness was assessed through a battery of performance tests, including measures of maximum muscular power, isometric strength, local muscular endurance, agility, and aerobic capacity. To avoid fatigue that could compromise the performance in the tests, data collection followed the following sequence, based on McGuigan’s proposal [27]: anthropometric measurements, maximal muscular power, maximal isometric strength, agility, local muscular endurance, and aerobic capacity. During all tests, verbal encouragement was provided in order that the best performance be achieved.

Maximal muscular power was assessed by standing long jump test [28]. Runners stayed behind a take-off line with feet slightly apart, and after a countermovement of swinging their arms and bending their knees, they jumped as far as possible. Jump-length was defined as the largest distance from the take-off to the nearest point of landing. All participants performed three attempts with a recovery of two minutes between them, and the best score was considered as the personal result.

Maximal isometric strength was determined through handgrip test. Test consists in squeezing the hydraulic dynamometer (Jamar^®^) as strong as possible for 3–5 s in a sitting position, with the elbow flexed at 90° [29,30]. Participants practiced the use of the equipment before the test. Participants performed the test twice (30 s apart) for each hand. The mean value of the best result for each hand was used as the personal result, and then it was adjusted for participant’s body weight (and this ratio was used in further analysis).

For muscular endurance performance evaluation, participants accomplished sit-up and push-up tests. The sit-up test assessed abdominal muscle endurance. Subjects lay on a soft mattress on their back with their knees flexed at 90° and hand/arms interlaced behind the head. An evaluator held both participant’s feet to the floor during the test. After the starting command, the subject raised his shoulders from the ground towards his knees, while keeping hands behind his head. The crunch movement ended when the elbows touch the knees. Immediately after, the subject returns to the starting position, until the shoulders make contact with the ground. Results are expressed as the maximal valid number of repetitions performed in 60 s [31]. The push-up test assessed upper-body muscular endurance. Participants assumed the standard push-up starting position, placing hands shoulder-width apart while keeping elbows and body straight. Repetitions were counted only if the upper arms flex elbows until achieving a position parallel to the ground and then back to the starting position. Participants had to perform the maximal number of push-up repetitions in 60 s [28]. 

The shuttle-run agility test estimated runners’ change of direction ability. Participants ran between two lines, 9.14 m apart, to pick up two small blocks (one at a time), as fast as possible. Subjects performed the test twice (1 min recovery), and the best result (lower elapsed time) was registered (in seconds and hundredths of a second) [32].

Cardiorespiratory fitness and running pace were estimated through the Weltman test [33], where participants had to run 3200 m on an outdoor 400 m track, as fast as possible. Time spent to cover the distance was registered by a chronometer (Vollo, model VL1809, São Paulo, Brazil), and the running pace was obtained from the equation (time (min)/3.200 m). In addition, according to suggested by Weltman et al. [33], the VO_2peak_ was estimated, and used as an indicator of cardiorespiratory fitness. 

Runners performed the Weltman test wearing a pedometer (Omron, Model HJA, São Paulo, Brazil), with the purpose of estimating their step frequency during the test. With this information, it was possible to estimate their stride frequency (steps/min). 

Results from each physical fitness test were transformed in z-scores (except the cardiorespiratory fitness, since this variable was only used to characterize the sample, and it was not included in the analysis), and after that a score of global physical fitness was computed by the sum of all physical fitness tests z-scores. Due to the inverse relationship between the result (time in seconds) and performance status, agility z-score was multiplied by (−1).

### 2.6. Data Quality Control

The data quality control was performed in two steps. Firstly, under the supervision of an expert in anthropometric measurements, a pilot study was conducted with five runners, which were measured twice for all the anthropometric variables. In addition, those runners were also evaluated for all the physical fitness tests, allowing to train the procedures for all data collection. Secondly, during data collection, each runner was evaluated twice for all the anthropometric variables, and a third measurement was performed if the difference between the first two measurements was higher than the tolerance values adopted: 0.5 kg for weight; 0.5 cm for height and for sitting height. All assessment was conducted for only two evaluators.

### 2.7. Statistical Analysis

Data was expressed as mean (standard deviation) and median (interquartile range) or frequency (%). All the variables were tested for the normality by the Shapiro-Wilk test. Aiming to describe differences in global physical fitness based on running performance, the sample was split into tertiles according to running pace. First tertile was called as “semi-professional runners” (pace ≤ 266 s/km), the second tertile was labelled as “amateur runners” (pace between 267–308 s/km), and the third tertile was named as “recreational runners” (pace > 308 s/km). An independent-sample Kruskal-Wallis test, followed by U Mann-Whitney (with adjustment for the *p*-value), was computed to identify differences between runners’ groups for descriptive variables. Correlations (Pearson or Spearman, based on variables distribution) between dependent and independent variables were computed. Variables that were not significantly correlated with running pace (upper-body muscular endurance) were not included in further analysis. A linear regression analysis, with predictors hierarchically introduced into the models, was used to determine predictors associated with running performance. Previously, the assumptions of multivariable regression (such as normality of residuals, homoscedasticity, multicollinearity) were tested, and none of them were violated. Running pace was used as dependent variable, and associated factors were inserted in three models, increasing the complexity level: model 1–biological variables (age and body fat); model 2–model 1 and physical fitness (agility, maximum muscular power, isometric strength, local muscular endurance, and global fitness) and stride frequency; model 3–model 2 and training variables (volume, frequency training/week, practice time). All analysis was conducted in SPSS (IBM, version 24.0, Armonk, NY, USA), with a 5% significance level.

## 3. Results

As a whole, runners showed a mean age of 36.9 ± 12.5 years and 19 ± 6% of body fat. Furthermore, the semi-professional runners presented the best mean values for running pace and the highest training volume/week, when compared against amateur and recreational groups. A great variability within and between groups for each physical fitness component test was observed, based on results presented on Table 1. For the biological variables, a significant difference was observed between groups, where recreational runners showed to be the oldest, the heaviest, and those with the highest fat percentage. It was not found statistically significant differences for upper-body muscular endurance, abdominal muscular endurance, isometric strength, and stride frequency between groups.

**Figure 1:**
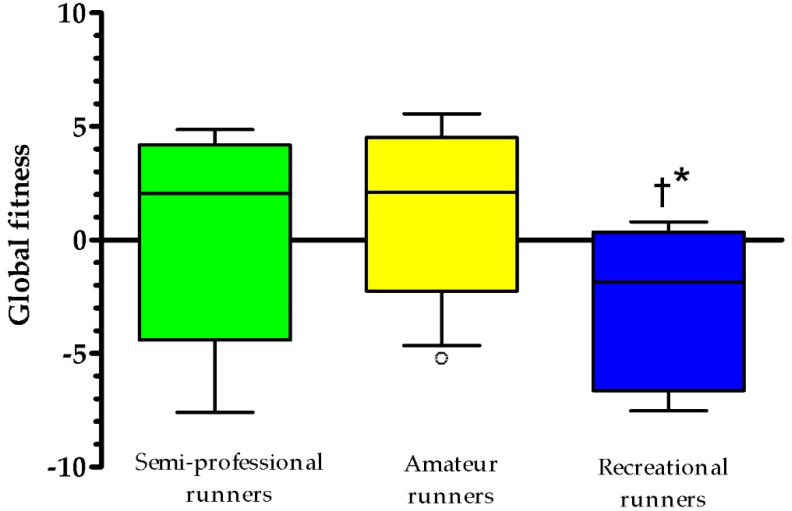
Differences in global physical fitness among runners’ groups, based on performance. † indicates significant difference when compared against semi-professional runners; * indicates significant difference when compared against amateur runners; circle indicates outliers.

Statistically significant differences were observed in global fitness score between runners’ groups (H(2) = 8.78, *p* = 0.01). Recreational runners showed the lowest global physical fitness score when compared against semi-professional runners (U = 10.00; *p*-adjusted = 0.04) and amateur runners (U = 11.59; *p*-adjusted = 0.01) (Figure 1).

Further, to estimate predictive power of variables studied, a regression model was built. Model 1 from the linear regression shows that body fat% was significantly associated with a slower running pace (β = 7.27; 95% CI = 4.00–10.55). When physical fitness components were included (Model 2), age showed to be a significant predictor, meaning that increasing age the running performance decreases (β = 1.79; 95% CI = 0.22–3.35). Regarding the physical fitness components, only agility was not significantly associated with the performance. Muscular power, isometric strength, abdominal muscle endurance, and stride frequency were directly associated with performance (higher values on these components, lead to decreasing time to cover one kilometer); however, the global fitness was inversely associated with the performance (β = 28.43; 95% CI = 12.76–44.09). In the final model (Model 3), biological and training variables did not show to be associated with the running performance, and the physical fitness components (significantly related to it on model 2) remained to be significantly related. This final model explains 86% of the total variance in running performance (Table 2).

## 4. Discussion

The rise in the number of running events around the world has led to an increase in interest to understand which factors would be associated with the performance in their modality. It seems that there is some consensus that performance derives from a set of variables related to body components, athletic health, injury risk, socioeconomic variables, and environmental factors [34,35]. Taking this into account, the present study investigated the role of biological, physical fitness, and training variables in the prediction of running performance, observing that variables included in our final regression model explained 86% of the variance in the performance in the sample studied.

In the first model, with only biological variables included, body fat% showed to be negatively associated with the performance, and this result is in agreement with previous studies [36,37,38]. In the study of Maciejczyk et al. [37], runners with higher body fat% presented the smallest VO_2máx_, while Ghiani et al. [38] and Longman et al. [39] found that increases in body fat% rises heat production, also increasing the energy cost during running. These factors demand adjustments in stride length and/or frequency, leading to a decrease in the performance.

When only biological and physical fitness variables were considered (model 2), older runners presented worse running performance than their youngest peers (increasing age, decreases the running performance). This result can be associated with physiological (decrease of cardiovascular function and strength) and biomechanical factors, as well as lifestyle/behaviors (e.g., work, family, training commitment) that can influence in runners’ performance, especially after becoming 35 years of age (masters athletes) [40,41]. Regarding physical fitness components, muscular power, abdominal muscular endurance, and isometric strength presented a direct association with running pace. The strength required to move the body while running is the primary component of energy expenditure. Thus, a higher lower limb power would facilitate horizontal displacement, decreasing chances of injuries due to muscle imbalances, reducing muscle damage [42], and improving running economy [43,44,45]. In addition, greater abdominal muscular endurance can also favor running efficiency, given that previous studies reported that intervention focusing on the improvement of the core muscles provided better running economy, higher force production in the leg movement control during running activity, and reduction in the risk of injuries [18,45]. 

Moreover, it was found that a higher stride frequency decreases the time spent to cover 1 km. Although there is no consensus, this may occur since a higher stride frequency shortens the time of ground contact, resulting in a more economic running [46,47]. Expert runners usually have higher step rates and shorter stride length than the novice ones, aiming to optimize energy expenditure and to reduce injury risk–in a mechanism called “self-optimization” [46,48,49,50,51]. Present results can be explained by the large variability of runners’ practicing time among studied subjects.

Alongside the models, an inverse association between global fitness score and runners’ pace was observed. When the sample was stratified in tertiles, according to runners’ pace, a significant difference between groups for global fitness score was observed, where “recreational runners” presented the lowest mean value, and this result was statistically different from their peers. In addition, “amateur runners” had the highest mean value for global fitness score, and despite no difference be observed when compared to “semi-professional runners”, we speculate that “amateur runners” may be engaged not only in specific running training practices, but they may be involved in additional training program to develop other fitness variables [52]. On the other hand, “semi-professional runners” may have a greater focus on developing skills they judge are directly related (and more important) to running (e.g., aerobic capacity) [20]. This idea is in agreement with previous researches, where the fastest marathon runners did not present the highest anaerobic power (when compared to values from the general population) [13,53]. Since this physical fitness component was not directly related to performance in marathon runners, it is possible that the best runners do not “spend their time” training capabilities that, apparently, do not significantly improve running pace. 

Despite the results reported in the present study, we advocate that the development of global fitness should be considered in running training programs, given that athletes are exposed to several constraints (e.g., changes in surfaces characteristics and inclination) that can impose a high involvement of anaerobic metabolic pathway [7]. Further, anaerobic components have been suggested as relevant variables to differentiate athletes with similar values of aerobic capacity [15], and also as indicators of fatigue levels among athletes [54,55], meaning that these variables can contribute to improvement in runners’ performance, leading to better results. In addition, we postulate that our results could have been influenced by results achieved by the “amateur runners group”, since they had the best results for the global fitness. Furthermore, while “semi-professional runners” had presented a lower global physical fitness, they also presented a higher variance in this variable, meaning that there are inter-individual differences within this group, highlighting they maybe were not interested in train and develop global physical fitness as a whole, focusing on those components most related to their practice/performance. Taking the abovementioned idea, it is possible that those “semi-professional” runners who improve their global fitness, can have an advantage against their peers with similar aerobic capacity, but with lowest global physical fitness. 

Unlike observed in previous researches [14,21,56], in the present study, training variables were not a significant predictor for running pace. These results can be associated with aspects related to social and built environments (e.g., socioeconomic conditions, place of residence, available places to practice), that can influence training practice [57,58] and, as a consequence, performance. Variables derived from the environment were not explored in the present study, so we are just able to speculate about possible explanations for this result. 

This study has some limitations. Information related to the environment where participants were encompassed, or belong to, was not taken into account. As much as it might reveals further details about the characteristics of the sample and training conditions, this study was thought to focus on easy-to measure variables (potentially related to performance), as well as be more susceptible to change. Also, the identification of running pace considering only the average pace during the 3200 m test. It is possible that runners use different pace strategies during races [59], especially slower runners, who tend to present the highest pace variation [60]. However, field tests seem to be accurate enough to predict long distance running performance [61,62]. Another point to be highlighted as a limitation is related to the fact that there was a heterogeneity regarding runners regularly covered distance, or race events preference (e.g., 5 km, 10 km, half-marathon, marathon, ultramarathon), which limits the generalization of the results. In addition, the sample size is another point to be mentioned. Notwithstanding the sample size be previously computed, it was not possible to reach it due to the COVID-19 pandemic, which forced the suspension of the data collection. However, despite the small sample size, all of the assumptions related to the statistical analysis used were met. On the other hand, some strengths of the study must be mentioned, such as the fact that all measurements were conducted using instruments and protocols accessible for coaches, trainers, and exercise physiologists, in order to respect ecological validity. The global fitness score was also a simple methodological approach that allowed to summarize a set of performance-related variables, and that could be probable customized for particular purposes. Besides, global fitness score stratified accurately runners by level of performance.

## 5. Conclusions

From the set of the investigated variables, individual characteristics and physical fitness components showed to be significant predictors for running performance. Considering that performance is a multifactorial phenomenon, the marginal gains concept (the idea that hidden advantages, when summed, can improve the athlete’s performance) seems to be relevant and must be considered when focusing in better sports results. In this sense, even physical fitness components which are usually not considered as relevant to runners’ performance (such as isometric strength), must be trained aiming to improve running pace, since its effect on the performance, when associated with cardiorespiratory fitness and lower limb strength, for example. Given that, we encourage coaches to develop the runners’ global fitness, instead of focusing only on fitness components thought to be more relevant for running practice, which can lead to improvement in the performance.

## Figures and Tables

**Table 1:** Descriptive statistics [mean (standard deviation) and median (interquartile rage); or frequency] of the sample.

	Semi-Professional Runners	Amateur Runners	Recreational Runners	Semi-Professional Runners	Amateur Runners	Recreational Runners
Variables	Mean (Standard Deviation)			Median (Interquartile Range)		
*Biological variables*						
Age (years)	30 (9.3)	35. (8.7)	46 (14.1)	29 (13)	34 (6)	49 (36) †
Body mass (kg)	65.2 (8.7)	74.6 (5.2)	78.5 (13.3)	71.3 (17.1)	74.1 (11.3)	79 (24.5) †
Body fat (%G)	14.3 (5)	19.2 (4.2)	24.9 (3.9)	14.89 (11.0)	18.75 (8.51)	23.8 (5.9) †
*Physical fitness*						
VO_2_Peak (mL·kg^−1^·min^−1^)	58.51 (7.0)	46.4 (3.2)	33.0 (15)	55.7 (10.6)	47.4 (6.1) †	35.3 (19.8) †
Agility (s)	10.9 (1)	10.8 (0.8)	12.3 (0.9)	10.84 (1.0)	10.7 (1.0)	12.2 (2) †,*
Muscular power (cm)	181.2 (39.8)	199.1 (30.9)	151.5 (25.4)	191 (34.6)	197.5 (43.4)	148.6 (43.8) †,*
Upper-body muscular endurance (rep)	32.3 (12.6)	35.08 (10.0)	29.2 (10.4)	31 (17)	35.5 (20)	27.5 (21)
Abdominal muscular endurance (rep)	35.5 (10.2)	32.8 (6.2)	23.7 (12.4)	32 (18)	30 (7)	26 (17)
Isometric strength (kg)	0.60 (0.11)	0.58 (0.89)	0.53 (0.86)	0.62 (0.15)	0.59 (0.16)	0.53 (0.14)
Stride frequency (steps/min)	173.7 (7.0)	177.2 (9.2)	167.4 (10.6)	176 (11.43)	177 (11.62)	168.7 (19.9)
Global fitness	0.66 (4.0)	1.49 (2.7)	−2.8 (3.0)	1.90 (4.24)	1.76 (2.85)	−1.85 (5.64) †,*
*Training variables*						
Practice time (months)	82.7 (68.9)	68.4 (44.9)	83.09 (93.3)	84 (71)	60 (87)	48 (60)
Running pace (s)	235.5 (27.6)	283.8 (12.3)	355.5 (50.2)	247 (42)	279 (19)	343 (57)
Volume/week (km)	46.7 (35.6)	33.3 (18.3)	24.3 (13.3)	30 (48)	37.5 (31)	22 (10)
*Training frequency*			**Frequency (%)**			
≤3 sessions/week	4 (33.3%)	5 (41.7%)	10 (90.9%)			
>3 sessions/week	5 (41.70%)	6 (41.7%)	1 (9.10%)			
Missing information ^¥^	3 (25%)	2 (16.7%)	2 (16.7%)			

Isometric strength adjusted for body mass; † significantly different when compared to semi-professional runners group; * significant different when compared to amateur runners group; ^¥^ missing data from all the training variables, except for running pace.

**Table 2:** Results of Linear Regression for predictors in running performance.

Variables	Model 1 Biological Variables		Model 2 Physical Fitness		Model 3 Training Variables	
	β	95% CI (Lower–Upper)	β	95% CI (Lower–Upper)	β	95% CI (Lower–Upper)
*Constant*	175.06	110.07–240.06	1212.87	810.29–1615.43	1191.94	774.11–1609.76
Age (years)	−0.66	(−2.30)–0.99	1.79 *	0.22–3.35	1.56	(−0.08)–3.20
Body fat%	7.27 *	4.00–10.55	2.90	(−0.13)–5.94	2.16	(−1.18)–5.51
Agility (s)			7.12	(−17.25)–1.48	8.90	(−16.86)–34.66
Muscular power (cm) Isometric strength			−1.13 * −380.80 *	(−1.72)–(−0.52) (−617.05)–(−144.54)	−1.02 * −422.95 *	(−1.69)–(−0.35) (−689.65)–(−156.25)
Abdominal muscle endurance (rep)			−5.00 *	(−7.40)–(−2.59)	−4.81 *	(−7.52)–(−2.10)
Global Fitness			28.43 *	12.76–44.09	27.14 *	9.52–45.03
Stride frequency			−3.14 *	(−4.38)–(−1.89)	−2.99 *	(−4.29)–(−1.69)
Time practice (months)					0.13	(−0.08)–0.33
Frequency/week					−0.18	(−16.31)–15.95
Volume (km/week)					−0.29	(−1.41)–0.83
R^2^	0.47		0.84		0.86	

*: *p* < 0.05; 95% CI: 95% confidence interval.

## Data Availability

The data are not publicly available due to ethical concerns.
